# Predictors of First Anti-TNF Treatment Failure in Patients with Inflammatory Bowel Disease: A Single-Center Cohort Study

**DOI:** 10.3390/biomedicines14050984

**Published:** 2026-04-24

**Authors:** Konstantinos C. Mpakogiannis, Paraskevi Chasani, Ioanna Nefeli Mastorogianni, Konstantinos H. Katsanos, Fotios S. Fousekis

**Affiliations:** 1 Division of Gastroenterology, Department of Internal Medicine, Faculty of Medicine, School of Health Sciences, University of Ioannina, 45110 Ioannina, Greece; kostismpakogiannis@gmail.com (K.C.M.);; 2 Institute of Neuropathology, Universitätsklinikum Erlangen, Friedrich-Alexander-Universität Erlangen-Nürnberg, 91054 Erlangen, Germany; 3 Medical Department 2, Academic Teaching Hospital Fürth, Friedrich-Alexander Universität Erlangen-Nürnberg, 90766 Fürth, Germany

**Keywords:** anti-TNF, Crohn’s disease, ulcerative colitis, treatment failure, predictors

## Abstract

**Introduction**: Despite proven efficacy of anti-TNF agents in inflammatory bowel disease, primary non-response affects up to one-third of patients, while secondary loss of response occurs at 13–21% per patient-year, often requiring dose optimization or switching to alternative advanced therapies. **Methods**: The present single-center cohort study at the University Hospital of Ioannina included biologic-naïve patients receiving anti-TNF therapy as their first biologic treatment. First anti-TNF treatment failure was defined as discontinuation due to persistent IBD activity despite maximal dose optimization (infliximab 10 mg/kg every 4 weeks, adalimumab 40 mg weekly). Patients with measurable anti-drug antibodies prior to anti-TNF dose intensification or discontinuation were excluded. Of 528 anti-TNF-treated patients, 286 (173 with CD, 113 with UC) met the inclusion criteria and were included in the final statistical analysis. **Results**: Anti-TNF failure occurred in 32.7% of Crohn’s (CD) and 32.9% of ulcerative colitis (UC) patients. Multivariable Cox regression identified complicated phenotype (stricturing or/and penetrating CD; HR = 1.9, *p* = 0.032) and concomitant corticosteroid use at anti-TNF initiation (HR = 2.03, *p* = 0.012) as independent predictors of anti-TNF failure in CD. Age at CD diagnosis showed a trend for statistical significance (HR = 1.02, *p* = 0.061), and after stratification, age at diagnosis ≥ 40 years conferred higher risk (HR = 1.93, *p* = 0.016), alongside persistent effects of complicated phenotype (HR = 1.83, *p* = 0.027) and corticosteroid use (HR = 2.01, *p* = 0.013). In UC patients, female sex predicted anti-TNF failure (HR = 2.13, *p* = 0.025). IBD-related bowel resection occurred in 26.6% of patients with CD and in 5.3% of patients with UC. **Conclusions**: Anti-TNF failure remains common despite optimization. Identifying immunogenicity-independent predictors may enable personalized treatment strategies and improve outcomes.

## 1. Introduction

Inflammatory bowel diseases (IBD) comprise a group of chronic, immune-mediated inflammatory disorders of the gastrointestinal tract, encompassing Crohn’s disease (CD) and ulcerative colitis (UC) [1]. CD disease can affect any segment of the gastrointestinal tract, from the oral cavity to the anus, and is characterized by transmural inflammation and a discontinuous pattern of involvement [1]. In contrast, UC is limited to the colon and rectum, with inflammation confined to the mucosal layer and typically presents in a continuous manner [1].

Given their chronic relapsing-remitting course and progressive nature, IBD is associated with substantial morbidity and a significant impairment in patients’ quality of life [2]. Disease-related complications, including strictures, fistulas, recurrent hospitalizations, and the need for surgical intervention, are common, particularly in cases of suboptimal disease control [3]. Consequently, early and effective therapeutic strategies aimed at inducing and maintaining remission, preventing complications, and improving long-term outcomes represent a cornerstone of contemporary gastroenterological practice [4].

The therapeutic landscape of IBD underwent a major transformation with the introduction of biologic agents in the late 1990s [5]. Infliximab, a chimeric monoclonal antibody targeting tumor necrosis factor alpha (TNF-α), was the first biologic therapy approved for IBD and the first anti-TNF agent used in this patient population [5]. Subsequently, additional anti-TNF agents, including adalimumab, certolizumab-pegol, and golimumab, were introduced into clinical practice [6]. Among these, infliximab and adalimumab remain the most widely prescribed anti-TNF agents worldwide, supported by robust efficacy data, long-term safety profiles, and extensive clinical experience [7,8].

Despite their established efficacy, approximately 30% of patients with IBD fail to achieve an initial therapeutic response to anti-TNF therapy, reflecting primary non-response [9]. Moreover, secondary loss of response to anti-TNF therapy following successful induction of remission occurs at an estimated rate of 13–21% per patient-year, frequently necessitating dose optimization or switching to alternative advanced therapies [10].

Therefore, the identification of prognostic factors associated with anti-TNF treatment failure and the subsequent need for anti-TNF therapy discontinuation is of critical clinical importance [11,12].

Accordingly, we conducted a single-center, real-world cohort study at a tertiary referral center for the monitoring and management of patients with IBD in Epirus and Northwestern Greece. The primary objective of this study was to investigate potential predictors of first anti-TNF treatment failure. The secondary outcome of the study involved the proportion of patients requiring IBD-related bowel resection following the initiation of the first anti-TNF agent.

## 2. Materials and Methods

### 2.1. Patients

Patients with histologically confirmed IBD, treated with Infliximab or adalimumab, were included in this single-center cohort study. Clinical and demographic data for each patient were obtained retrospectively and/or prospectively from medical records and the hospital’s electronic database between October 2023 and July 2025. A combined retrospective and prospective follow-up of patients was conducted until the end of July 2025. It should be mentioned that patients with unclassified IBD, as well as patients treated with golimumab or certolizumab-pegol were excluded from the study due to small sample sizes (8 patients).

### 2.2. Definitions

Treatment failure was defined as discontinuation of anti-TNF therapy due to persistent or recurrent IBD activity (as determined by the treating physician’s clinical judgment) despite optimization to the maximum feasible dose [13,14]. The absence of measurable anti-drug antibodies prior to anti-TNF dose intensification and anti-TNF discontinuation was essential. Maximal dose escalation was 10 mg/kg every 4 weeks for infliximab and for adalimumab, preferably 40 mg weekly. Other reasons for anti-TNF withdrawal were categorized as potential anti-TNF-related adverse events, patient preference, clinical remission, and pregnancy. Data from endoscopic procedures performed up to three months prior to anti-TNF initiation were extracted from endoscopy reports and considered as baseline characteristics in the analysis of predictors of first anti-TNF treatment failure. Short-term use of immunomodulators or corticosteroids (<30 days) was disregarded. Baseline immunomodulator or corticosteroid use was defined as either initiation within 30 days before or continued administration for at least 30 days after anti-TNF initiation. Anti-TNF treatment interruptions of less than three months were allowed.

### 2.3. Inclusion and Exclusion Criteria

Patients were eligible for inclusion in the study if they met all of the following inclusion criteria:

(1) IBD diagnosis is based on clinical, laboratory, endoscopic, and histological findings.
(2) Minimum follow-up period of 6 months after the first anti-TNF therapy initiation.
(3) Treatment with an anti-TNF agent was administered in the Department of Gastroenterology at the University Hospital of Ioannina.
(4) Biologic-naïve status prior to the first anti-TNF agent initiation.
(5) Absence of anti-drug antibodies before anti-TNF treatment dose escalation and anti-TNF discontinuation. This exclusion focused on identifying potential immunogenicity-independent potential predictors of anti-TNF treatment failure.

Patients were excluded if any of the following criteria applied:

(1) Failure to attend, for any reason, scheduled follow-up visits at the Department of Gastroenterology or the outpatient clinic.
(2) Presence of measurable anti-drug antibodies prior to dose intensification or before the first anti-TNF treatment discontinuation.
(3) Non-biologic-naïve status prior to first anti-TNF agent initiation.
(4) Discontinuation of first anti-TNF therapy due to potential anti-TNF-related adverse effects, patients’ wishes, clinical remission, or pregnancy.

### 2.4. Statistical Analysis

Patient characteristics were expressed as absolute and relative frequencies for categorical variables and as medians along with interquartile range (q1, q3) for continuous variables. Univariate and multivariate Cox regression analyses were conducted to identify potential predictors of anti-TNF treatment failure. Variables with a *p*-value < 0.1 in the univariate analysis were included in the multivariate analysis. Hazard ratios (HRs) and 95% confidence intervals (CIs) were reported. A *p*-value ≤ 0.05 in the multivariate analysis was considered statistically significant. Time-to-event outcomes were analyzed using Kaplan–Meier curves with 95% confidence intervals (CIs). All statistical analyses were performed using SPSS software (version 29.0.2) and R (version 4.5.0; R Foundation for Statistical Computing, Vienna, Austria).

### 2.5. Study Flowchart

**Figure 1:**
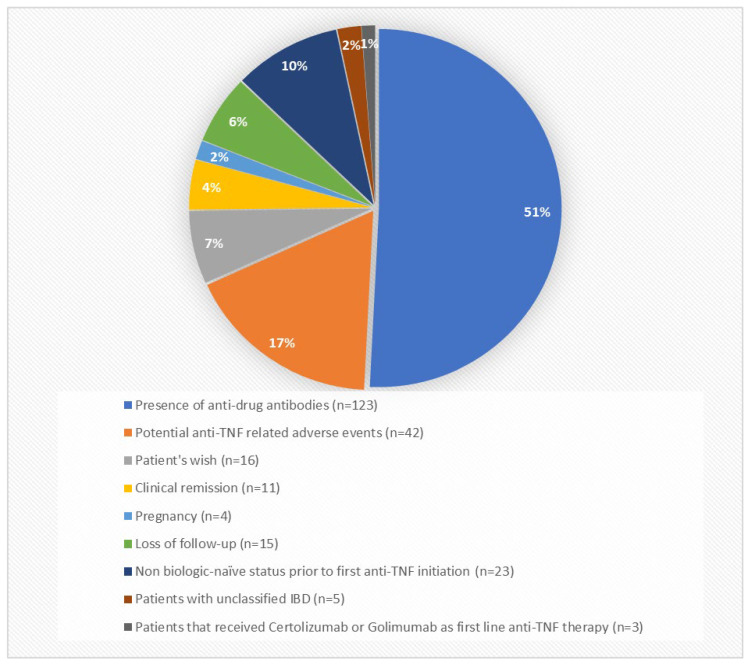
Reasons for patient exclusion from the final statistical analysis.

**Figure 2:**
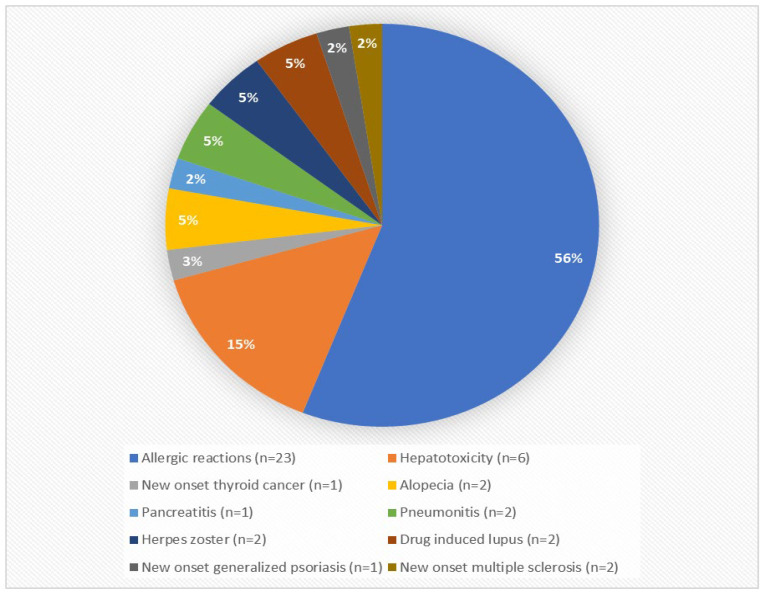
Potential Anti-TNF-related adverse events.

A total of 528 patients were initially included in the present cohort study. A total of 242 patients were excluded from the final statistical analysis as they met one of the aforementioned exclusion criteria. Specifically, 15 patients excluded due to loss of follow-up observation, 23 patients because they were not biologic-naïve prior to first anti-TNF agent initiation, 5 patients that diagnosed with unclassified IBD, 3 patients that received certolizumab or golimumab, while 123 discontinued anti-TNF agent due to measurable anti-drug antibodies, 42 patients discontinued due to potentially treatment-related adverse events, 16 patients withdrew from anti-TNF therapy due to their own preference, 11 patients discontinued biologic treatment due to clinical remission, and 4 patients due to pregnancy (Figure 1 and Figure 2).

**Figure 3:**
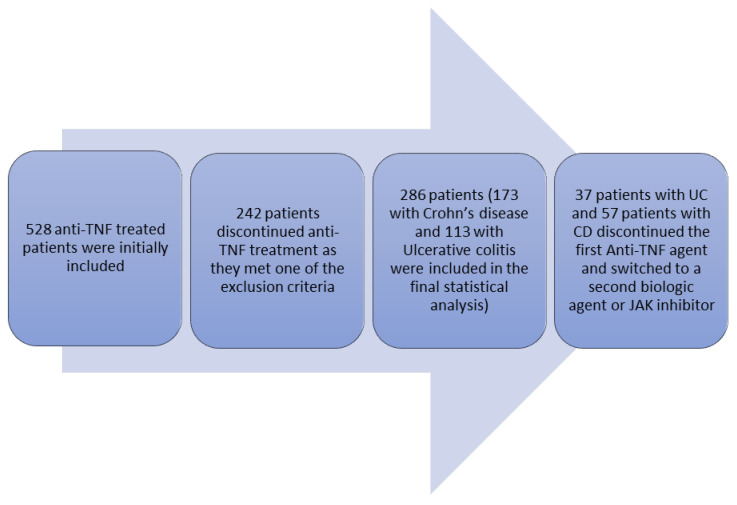
Study flowchart.

**Figure 4:**
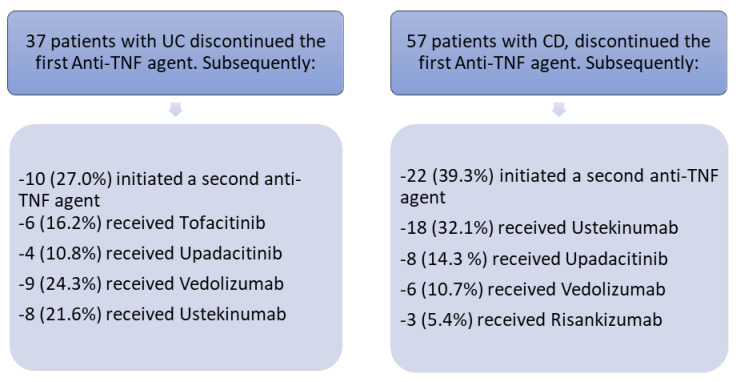
Second treatment choice after first anti-TNF treatment failure.

Ultimately, 286 patients were included in the final statistical analysis. During the follow-up period, 37 patients with UC (32.7%) and 57 patients with CD (32.9%) discontinued first-line anti-TNF therapy due to treatment failure. The study flowchart is illustrated in Figure 3. The second treatment choice after the first anti-TNF treatment is summarized in Figure 4.

## 3. Results

### 3.1. Cohort Characteristics

#### 3.1.1. Baseline Patients’ Characteristics

A total of 286 patients (173 with CD and 113 with UC) were included in the final statistical analysis, with comparable median follow-up durations (CD: 9.5 years; UC: 10 years). Among patients with CD, 42.2% were females, while the corresponding percentage among patients with UC was 40.7%. Other characteristics involved the family history of IBD (CD: 26.6%, UC: 30.1%), as well as laboratory parameters at anti-TNF initiation, including C-reactive protein (CD: 10 mg/L, UC: 9 mg/L), albumin (CD = 41 g/L, UC = 39 g/L), hemoglobin (CD: 13.1 g/dL, UC: 12.9 g/dL), leukocyte count (CD: 8610/μL, UC: 7960/μL), and platelet count (CD: 363,000/μL, UC: 305,000/μL).

#### 3.1.2. Baseline Disease Characteristics

Among CD patients, disease location at anti-TNF initiation included ileal (L1) involvement in 36.4%, colonic (L2) in 9.8%, and ileocolonic (L3) in 53.8%, with upper gastrointestinal involvement present in 12.1% of CD cases. The corresponding CD phenotypes were non-stricturing, non-penetrating (B1) in 49.7%, stricturing (B2) in 31.8%, penetrating/fistulizing (B3) in 18.5%, and perianal disease in 16.2%, while 11.0% had a history of bowel resection before anti-TNF initiation. Among UC patients, disease extent at anti-TNF initiation was proctitis (E1) in 12.4%, left-sided colitis (E2) in 35.4%, and extensive colitis (E3) in 52.2%, and pseudopolyps were present in 27.4% of patients.

#### 3.1.3. Treatment Characteristics

Patients with CD were diagnosed at a younger median age than those with UC (29 vs. 34 years), though this difference lacked statistical significance (*p* = 0.248). However, CD patients initiated the first anti-TNF therapy at a significantly younger age (35 vs. 42 years; *p* = 0.005) and after a shorter disease duration (0.9 vs. 2.2 years; *p* = 0.003). Median persistence (defined as the time between onset and discontinuation of the first anti-TNF treatment) on first anti-TNF therapy was longer among CD than UC patients (2.7 vs. 1.5 years), but this difference did not achieve statistical significance (*p* = 0.271). Infliximab was the predominant first anti-TNF agent for both CD (116/173, 67.1%) and UC (86/113, 76.1%) patients, with adalimumab administered in the remaining cases (CD: 32.9%; UC: 23.9%). Among patients who discontinued first-line anti-TNF therapy, median treatment duration did not differ significantly between adalimumab and infliximab, either among CD patients (2.7 vs. 2.3 years; *p* = 0.225) or UC patients (1.8 vs. 1.5 years; *p* = 0.91). Concomitant immunomodulator therapy at anti-TNF initiation was administered to 33.5% of CD patients and 29.2% of UC patients, while corticosteroid co-administration occurred in 20.2% of CD patients and 23% of UC patients. The aforementioned baseline demographic and clinical characteristics are summarized in Table 1.

### 3.2. Predictors of First Anti-TNF Treatment Failure

As already mentioned, during the follow-up period, 37 of 113 patients with UC (32.7%) discontinued anti-TNF due to treatment failure. The corresponding percentage in patients with CD was 32.9% (57 of 173 patients). To assess predictors of treatment failure, univariable and multivariable Cox regression analyses were conducted. Complicated phenotype (stricturing or/and penetrating CD) (HR = 1.9, *p*-value = 0.032) as well as the concomitant use of steroids at anti-TNF initiation (HR = 2.03, *p*-value = 0.012) were identified as independent predictors of anti-TNF failure among patients with CD. Female sex emerged as an independent predictor of anti-TNF therapy failure in patients with UC (HR = 2.13, *p*-value = 0.025). Additionally, it should be underlined that age at CD diagnosis showed a trend toward statistical significance as an independent predictor (HR = 1.02, *p* = 0.061), suggesting that older age at diagnosis may increase the risk of anti-TNF failure. The results of univariable and multivariable Cox regression analyses are summarized in Table 2 and Table 3.

The age at CD diagnosis was further stratified into two categories: <40 years and ≥40 years. Age at diagnosis ≥ 40 years emerged as an independent predictor of anti-TNF treatment failure (HR = 1.93, *p* = 0.016). Complicated CD phenotype (HR = 1.83, *p* = 0.027) and concomitant corticosteroid use (HR = 2.01, *p* = 0.013) at anti-TNF initiation also remained statistically significant predictors of first-line anti-TNF treatment failure after stratification for age at CD diagnosis. The results of univariate and multivariate analyses after stratification for age at CD diagnosis are summarized in Table 4.

### 3.3. Persistence on Anti-TNF Treatment

**Figure 5:**
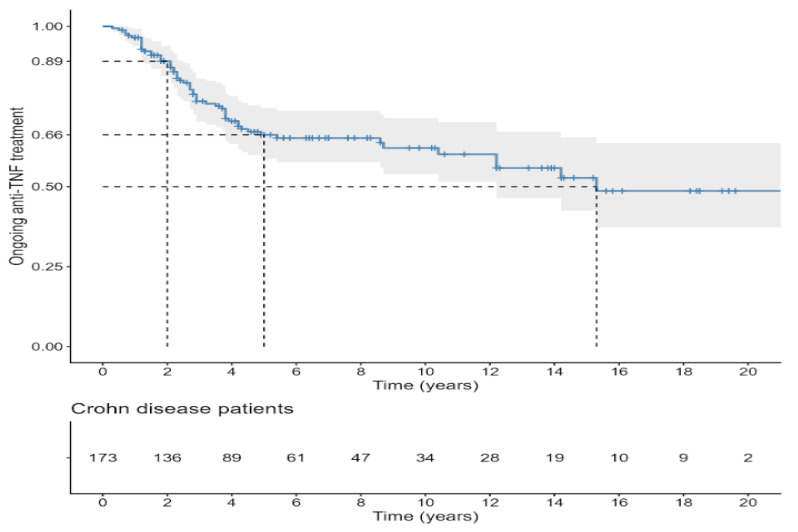
First anti-TNF treatment persistence in patients with CD.

**Figure 6:**
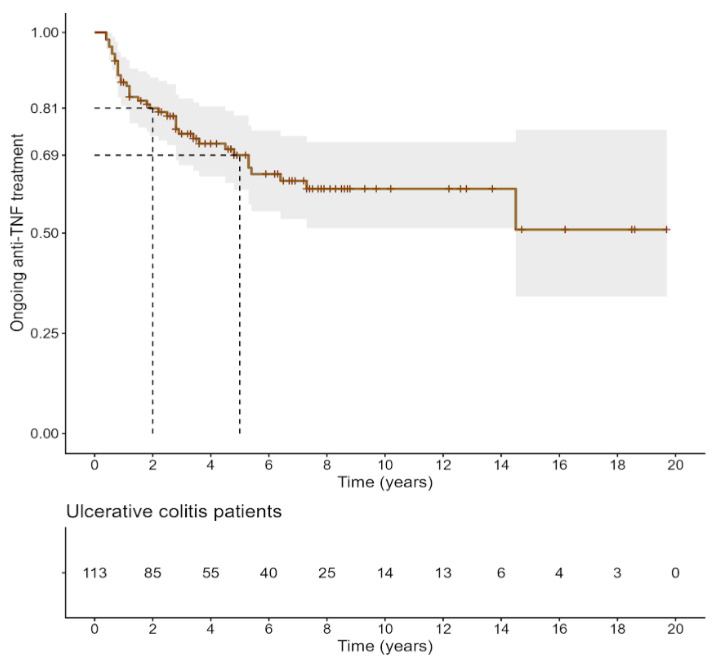
First anti-TNF treatment persistence in patients with UC.

Kaplan–Meier curves of first anti-TNF treatment persistence in patients with CD and UC are illustrated in Figure 5 and Figure 6, respectively. In both diseases, the probability of remaining on the initial anti-TNF agent declined progressively over time. In patients with CD, treatment persistence was approximately 89% at 2 years, 66% at 5 years, and around 50% at 15 years of follow-up. Similarly, in UC, persistence rates were about 81% at 2 years and 69% at 5 years, with nearly half of the patients remaining on therapy in the long term.

### 3.4. Secondary Outcome of the Study

Following initiation of the first anti-TNF agent, IBD-related bowel resection was required in 6 of 113 patients with UC (5.3%) and in 46 of 173 patients with CD (26.6%). Among patients with UC, four underwent bowel resection for colorectal cancer, while two required colectomies for acute severe UC refractory to medical therapy. Among patients with CD, three underwent bowel resection due to colorectal cancer (two patients) and small bowel cancer (one patient). In addition, 35 of 55 patients with stricturing CD (63.6%) required at least one bowel surgery at a median follow-up period of 10 years. In this subgroup of patients, the median time from anti-TNF initiation to surgery was 37 (8,94) months. In addition, eight patients with CD underwent bowel surgery due to other CD-related complications.

## 4. Discussion

In this real-world cohort study, approximately one-third of patients with CD and UC discontinued first-line anti-TNF therapy due to treatment failure. Our study demonstrates higher anti-TNF persistence rates than reported in the real-world literature. For CD, persistence was 89% at 2 years and 66% at 5 years, while for UC it was 81% at 2 years and 69% at 5 years, with nearly half of patients maintaining long-term therapy. Published cohort studies report lower persistence for CD (60–70% at 1 year, 40–50% at 2 years, 30–40% at 5 years) and UC (50–60% at 1 year, 30–40% at 5 years) [15,16,17]. The higher anti-TNF persistence rates in our cohort likely reflect methodological differences. We involved only biologic-naïve patients who discontinued first-line anti-TNF therapy due to treatment failure after maximal dose escalation and in the absence of detectable anti-drug antibodies, while excluding from the final statistical analysis anti-TNF treatment discontinuations due to adverse events, remission, patient preference, or pregnancy.

In patients with CD, a complicated phenotype at first anti-TNF therapy initiation was independently associated with anti-TNF treatment failure. This aligns with prior evidence demonstrating superior clinical outcomes, including sustained remission and long-term anti-TNF response rates in non-stricturing, non-penetrating CD compared to stenotic or fistulizing phenotypes [10,18,19,20,21,22]. TNF-α is a key pro-inflammatory cytokine that holds a central role in the pathogenesis of CD [23]. However, it remains challenging to separate the direct effects of anti-TNF therapy on fibrosis or fistula formation from its broader effect of reducing underlying inflammation [24,25]. Patients with fistulizing CD exhibit higher serum TNF-α levels and greater TNF-α expression in resected fistula tissues than individuals with non-fistulizing CD or controls, thereby reinforcing the rationale for anti-TNF therapy in penetrating CD [26,27]. Adegbola et al. performed a pilot study to measure anti-TNF drug concentrations in perianal fistula tract biopsies, contrasting samples from CD patients with those from idiopathic (cryptoglandular) fistulas [28]. Anti-TNF agents were detectable in idiopathic fistula tissues but were absent from all CD-associated fistula samples, implicating suboptimal tissue penetration as a key mechanism of anti-TNF failure in fistulizing CD [28]. Furthermore, while anti-TNF agents reduce TIMP-1 production in myofibroblasts by inhibiting TNF-α-mediated mesenchymal activation, which upregulates TIMP-1 and suppresses MMP-2 activity/collagen degradation, their ability to reverse established strictures in CD remains uncertain [29,30,31]. Furthermore, although early initiation of anti-TNF therapy has been associated with improved clinical outcomes in patients with fibrostenotic CD, the inflammatory component within fibrostenotic segments is less pronounced than in patients presenting with a purely inflammatory phenotype, a factor that may further limit anti-TNF efficacy in stricturing CD [18,21,32,33]. Concomitant corticosteroid use at anti-TNF initiation independently predicted first anti-TNF failure in CD patients. This likely reflects confounding by indication—steroid-dependent patients have more active, treatment-resistant disease inherently at higher risk of biologic failure, rather than direct corticosteroid effects on anti-TNF pharmacokinetics. Additional studies have emerged that corticosteroid use at anti-TNF initiation increases the risk of anti-TNF therapy failure, while also delaying therapeutic drug monitoring and optimization [34,35]. Notably, a recent meta-analysis revealed that corticosteroid use during anti-TNF induction in patients with CD was not associated with higher rates of clinical remission or response compared with patients not receiving corticosteroids, underscoring the potential absence of additive efficacy of corticosteroids in this therapeutic context [36]. Also, in our study, age at CD diagnosis showed a trend toward independent prediction of anti-TNF failure, with older age at diagnosis conferring a higher risk. After stratification, age at diagnosis ≥ 40 years was documented as an independent predictor of anti-TNF failure. These findings align with prior real-world data indicating that increasing age at diagnosis is an independent risk factor for reduced clinical benefit of infliximab and adalimumab in CD patients [37]. Indeed, older adults with new-onset inflammatory rheumatic diseases often have lower serum anti-TNF levels than younger patients, which may contribute to higher rates of anti-TNF treatment failure in this patient population [38]. However, other studies found no correlation between the age of CD onset and anti-TNF response [39,40].

Among patients with UC, female sex emerged as an independent predictor of anti-TNF treatment failure. Previous studies also emerged that women with rheumatologic diseases exhibited higher discontinuation rates of anti-TNF agents compared with male patients [41,42,43,44]. Notably, similarly to our study results, Blesl et al. identified female sex as an independent predictive factor for anti-TNF failure in patients with UC, a conclusion potentially attributable to augmented body fat and diminished hepatic clearance observed in females [17]. Nevertheless, other UC cohorts either documented male sex as a risk factor for anti-TNF failure or demonstrated no association between gender and anti-TNF failure [45,46,47,48,49]. These discrepancies highlight the need for further studies that will examine sex-based pathophysiological mechanisms and the effects of sex hormones on gastrointestinal inflammation and therapeutic response to anti-TNF agents [50].

A substantial proportion of patients with CD (26.6%) required IBD-related bowel surgery following anti-TNF initiation, compared with only 5.3% of those with UC. This disparity underscores the fibrostenotic and transmural nature of CD [51]. Notably, in patients with stricturing CD, surgery was required in nearly two-thirds, with a median interval of 37 months after anti-TNF onset, a finding that underscores the limited efficacy of current biologic therapies in preventing surgery in stenotic disease [52]. Cases of bowel resection due to IBD-associated colorectal cancer were rare (7 of 286 patients), a fact possibly supporting the chemopreventive role of current IBD medical therapies against colorectal cancer occurrence [53,54].

The present study has several methodological strengths. The study focused on clinical and therapeutic variables available at the onset of anti-TNF therapy, enabling the assessment of early predictors of response based on real-world data. Importantly, the exclusion of patients with detectable anti-drug antibodies prior to dose intensification or biologic discontinuation eliminated immunogenic failure bias. Moreover, treatment failure was defined as a loss of therapeutic efficacy after maximal dose optimization, ensuring that only cases of pharmacodynamic nonresponse were captured. Nevertheless, certain limitations must be acknowledged. The retrospective part of the study introduces potential risks of selection and regional bias. Also, a major limitation of our study is its single-center design, which may limit the generalizability of findings to other populations or healthcare settings with different patient demographics, disease management practices, or genetic backgrounds. Patients from our cohort may not fully represent the broader population of patients with CD or UC. Baseline fecal calprotectin levels and endoscopic activity assessment (Mayo score, CDAI) were not available, precluding additional assessment of inflammatory burden before anti-TNF initiation. Similarly, body mass index data and drug levels were absent, further limiting exploration of pharmacokinetic influences. Furthermore, the choice of the initial anti-TNF agent was partly determined by physician preference and drug availability within the national healthcare system. Similarly, discontinuation of the first anti-TNF agent was based on the clinical judgment of the treating physician, which introduces subjectivity into the results of our study. Despite these limitations, the present study provides valuable real-world evidence on potential predictors of first anti-TNF treatment failure, with findings from daily clinical practice and the treatment of patients with IBD.

## 5. Conclusions

Our observational findings emphasize the value of identifying clinical predictors of anti-TNF failure, particularly in the absence of anti-drug antibody formation. Recognizing patients who are at higher risk of anti-TNF treatment failure may help identify earlier therapeutic optimization through drug monitoring or switching to other biologics or JAK inhibitors. Future multicenter prospective studies are warranted to validate these predictors and refine personalized treatment approaches in IBD management.

## Figures and Tables

**Table 1:** Baseline demographic and clinical characteristics.

Characteristics	CD (n = 173)	UC (n = 113)
Median follow-up (years) (IQR)	9.5 (2, 20)	10 (5, 16.5)
Gender (number of females) (%)	73 (42.2%)	46 (40.7%)
Family history (first or second degree relatives) of IBD (%)	46 (26.6%)	34 (30.1%)
Smoking status at anti-TNF onset (%)	71 (41.6%)	29 (25.7%)
Median age at time of IBD diagnosis (years) (IQR)	29 (20, 42.5)	34 (22, 47.5)
Median age at time of anti-TNF onset (years) (IQR)	35 (23.5, 46)	42 (28, 58.5)
Median time from diagnosis until anti-TNF onset (years) (IQR)	0.9 (0.5, 3.7)	2.2 (0.55, 10.15)
Median persistence time of first anti-TNF biologic (years) (IQR)	2.7 (1.5, 3.9)	1.5 (0.8, 3.5)
Median C-reactive protein value at anti-TNF initiation (mg/L) (IQR)	11 (4, 36.5)	10 (4, 34)
Median Albumin value at anti-TNF initiation (g/L) (IQR)	41 (36, 44)	39 (31, 44)
Median Hemoglobin value at anti-TNF initiation (g/L) (IQR)	13.4 (12.2, 14.6)	12.9 (11.4, 14.7)
Median Leukocyte count at anti-TNF initiation (IQR)	8610 (4110, 11,590)	7960 (6320, 11,475)
Median Platelet count at anti-TNF initiation (IQR)	363,000 (256,000, 452,000)	305,000 (243,000, 401,000)
Concomitant use of steroids at anti-TNF initiation	35 (20.2%)	26 (23%)
Concomitant use of immunomodulators at anti-TNF initiation	58 (33.5%)	33 (29.2%)
Disease location CD at anti-TNF initiation (1) L1 (%) (2) L2 (%) (3) L3 (%)	(1) 63 (36.4%) (2) 17 (9.8%) (3) 93 (53.8%)	
Upper gastrointestinal involvement in CD (%)	21 (12.1%)	
CD phenotype at anti-TNF initiation: (1) B1 (%) (2) B2 (%) (3) B3 (%) (4) Perianal disease (%)	(1) 86 (49.7%) (2) 55 (31.8%) (3) 32 (18.5%) (4) 28 (16.2%)	
Number of patients with CD and a history of bowel resection before anti-TNF initiation	19 (11%)	
Disease location UC at anti-TNF initiation (1) E1 (%) (2) E2 (%) (3) E3 (%)		(1) 14 (12.4%) (2) 40 (35.4%) (3) 59 (52.2%)
Presence of pseudopolyps in patients with UC at anti-TNF initiation (%)		31 (27.4%)
First anti-TNF: (1) Infliximab (%) (2) Adalimumab (%)	(1) 116 (67.1%) (2) 57 (32.9%)	(1) 86 (76.1%) (2) 27 (23.9%)
Median persistence time of Adalimumab (years) (IQR)	2.3 (1.2, 3.7)	1.5 (1, 2.5)
Median Persistence time of Infliximab (years) (IQR)	2.7 (1.8, 4.4)	1.8 (0.7, 4.7)

**Table 2:** Univariable and multivariable Cox regression analysis of potential anti-TNF failure predictors in Crohn’s disease.

Variables	Univariate Cox Regression (Hazard Ratio) (95% HR) (*p*-Value)	Multivariate Cox Regression (Hazard Ratio) (95% HR) (*p*-Value)
Disease duration at anti-TNF initiation	HR = 0.95 (0.89–1.01), *p* = 0.111	
Age at CD diagnosis	**HR = 1.02 (0.99–1.03),** ***p* = 0.095**	HR = 1.02 (1–1.04), *p* = 0.061
Age at first anti-TNF onset	HR = 1.01 (0.99–1.02), *p* = 0.31	
Female sex Yes No (reference category)	HR = 1.23 (0.73–2.08), *p* = 0.43	
Family history of IBD Yes No (reference category)	HR = 1.33 (0.78–2.28), *p* = 0.30	
Smoking at anti-TNF onset Yes No (reference category)	HR = 1.51 (0.89–2.54), *p* = 0.123	
Concomitant use of steroids at anti-TNF initiation Yes No (reference category)	**HR = 2.04 (1.18–3.55),** ***p* = 0.011**	**HR = 2.03** **(1.17–3.53),** ***p* = 0.012**
Concomitant use of immunomodulators at anti-TNF initiation Yes No (reference category)	HR = 0.81 (0.35–1.89), *p* = 0.63	
Upper gastrointestinal involvement in CD Yes No (reference category)	HR = 1.38 (0.68–2.82), *p* = 0.375	
Complicated disease phenotype (stricturing or/and penetrating disease) Yes No (reference category)	**HR = 1.79 (1.06–3.5),** ***p* = 0.031**	**HR = 1.9** **(1.05–3.06),** ***p* = 0.032**
History of bowel resection before anti-TNF initiation Yes No (reference category)	HR = 1.12 (0.48–2.61), *p* = 0.791	
C-reactive protein levels at anti-TNF initiation	HR = 0.99 (0.98–1.00), *p* = 0.187	
Albumin levels at anti-TNF initiation	HR = 0.617 (0.329–1.156), *p* = 0.131	
Hemoglobin levels at anti-TNF initiation	HR = 1.15 (0.92–1.44), *p* = 0.215	
Leukocyte count at anti-TNF initiation	HR = 1 (1.00–1.00), *p* = 0.625	
Platelet count at anti-TNF initiation	HR = 1 (1.00–1.00), *p* = 0.154	

**Table 3:** Univariable and multivariable Cox regression analysis of potential anti-TNF failure predictors in ulcerative colitis.

Variables	Univariate Cox Regression (Hazard Ratio) (95% HR) (*p*-Value)	Multivariate Cox Regression (Hazard Ratio) (95% HR) (*p*-Value)
Disease duration at anti-TNF initiation	HR = 0.65 (0.95–1.03), *p* = 0.649	
Age at UC diagnosis	HR = 1 (0.99–1.02), *p* = 0.779	
Age at first anti-TNF onset	HR = 1 (0.98–1.02), *p* = 0.953	
Female sex Yes No (reference category)	**HR = 2.13 (1.1–4.12),** ***p* = 0.024**	**HR = 2.13** **(1.1–4.14),** ***p* = 0.025**
Family history of IBD Yes No (reference category)	HR = 1.15 (0.58–2.3), *p* = 0.687	
Smoking at anti-TNF onset Yes No (reference category)	HR = 1.06 (0.51–2.18), *p* = 0.886	
Concomitant use of steroids at anti-TNF initiation Yes No (reference category)	HR = 1.65 (0.86–3.2), *p* = 0.131	
Concomitant use of immunomodulators at anti-TNF initiation Yes No (reference category)	HR = 0.26 (0.03–1.92), *p* = 0.187	
Presence of pseudopolyps on recent endoscopy before anti-TNF initiation Yes No (reference category)	**HR = 1.84 (0.96–3.52),** ***p* = 0.064**	HR = 1.41 (0.7–2.86), *p* = 0.337
C-reactive protein levels at anti-TNF initiation	HR = 0.99 (0.98–1.01), *p* = 0.309	
Albumin levels at anti-TNF initiation	HR = 0.75 (0.41–1.38), *p* = 0.36	
Hemoglobin levels at anti-TNF initiation	HR = 0.93 (0.78–1.12), *p* = 0.440	
Leukocyte count at anti-TNF initiation	HR = 1 (1.00–1.00), *p* = 0.641	
Platelet count at anti-TNF initiation	HR = 1 (0.99–1.00), *p* = 0.293	

**Table 4:** Univariable and multivariable Cox regression analysis of potential anti-TNF failure predictors in Crohn’s disease after stratification for age at CD diagnosis.

Variables	Univariate Cox Regression (Hazard Ratio) (95% HR) (*p*-Value)	Multivariate Cox Regression (Hazard Ratio) (95% HR) (*p*-Value)
Age at CD diagnosis ≥40 years <40 years (reference category)	**HR = 1.77** **(1.05–3.02)** ***p* = 0.034**	**HR = 1.93** **(1.13–3.29)** ***p* = 0.016**
Concomitant use of steroids at anti-TNF initiation Yes No (reference category)	**HR = 2.04** **(1.18–3.55)** ***p* = 0.011**	**HR = 2.01** **(1.16–3.5)** ***p* = 0.013**
Complicated disease phenotype (Stricturing or/and penetrating disease) Yes No (reference category)	**HR = 1.79** **(1.06–3.5)** ***p* = 0.031**	**HR = 1.83** **(1.03–3.16)** ***p* = 0.027**

## Data Availability

The data presented in this study are available on request from the corresponding author.

## Abbreviations

The following abbreviations are used in this manuscript:

IBD	Inflammatory bowel diseases
UC	Ulcerative colitis
CD	Crohn’s disease
